# Clitoral cyst in a bitch

**DOI:** 10.1002/ccr3.2498

**Published:** 2019-10-23

**Authors:** Canny Fung, Jennifer M. Ortolani, Marc J. Greenberg

**Affiliations:** ^1^ BluePearl Veterinary Partners New York New York; ^2^ Critical Care Department BluePearl Veterinary Partners New York New York; ^3^ Surgery Department BluePearl Veterinary Partners New York New York

**Keywords:** canine, clitoral cyst, clitoral mass, clitoris, external genitalia, surgical resection, urinary incontinence

## Abstract

Clitoral cyst can be easily mistaken for a vaginal mass lesion and should be considered in the differential diagnosis for a female dog presenting with an anatomically abnormal external genital examination.

## Graphical abstract

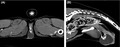

## 1 INTRODUCTION

The clitoris, vulva (pudendum femininum), and vestibule comprise the external genitalia of the female dog.^1^ The clitoris is the female homologue of the male penis. It is composed of paired roots, a body, and a glans and contains erectile tissue.^1^ Although the clitoris does not generally contain a bone, an osclitoridis may be present.^1^ An osclitoridis has been hypothesized to develop in response to natural endocrine disturbances, or altered hormone balance, such as alterations in androgen or progestin levels.^1^ Clitoral abnormalities in dogs are typically related to disorders of sexual development or masculinized conditions.^2^ Clitoral hypertrophy, development of an osclitoridis, and clitoral neoplasia have been described in female dogs.^2^ Additionally, male pseudohermaphroditism and XX sex reversal can lead to the development of a clitoris in male dogs.^2^

In humans, clitoral hyperplasia, cysts, and neoplasms have been described and can either be developmental or acquired.^3^ The majority of vulvar and clitoral cysts are reported secondary to female genital mutilation, and although many patients are asymptomatic, when enlarged, these cysts may cause pain.^4^, ^5^, ^6^

Clitoral cysts have not been reported in the veterinary literature. This case report details the diagnosis and treatment of a dog with an epithelial lined cyst arising from the clitoris.

## 2 CASE SUMMARY

A 10‐year‐old, female, spayed Australian Shepherd, weighing 26.1 kg, was presented to the primary care veterinarian for evaluation of urinary incontinence of 2 weeks duration. The owners reported that the patient would intermittently leak urine while laying down or getting off of the bed. Physical examination was unremarkable, with the exception of an enlarged and swollen vulva with a possible mass. A complete blood count and serum chemistry profile were normal. The urine specific gravity (Refractometer, Jorvet; Jorgensen Laboratories Inc) was 1.026, pH was 8.0, there were 4‐10 struvite crystals per high‐powered field (HPF) on sediment examination, and the remainder of the urinalysis and urine microscopy examination were unremarkable. A urine culture collected via cystocentesis was negative for bacterial growth. Abdominal radiographs were unremarkable. Based on the physical examination findings and diagnostic results, the patient was referred for a surgical consultation and further diagnostic evaluation.

Previous medical history included treatment for a urinary tract infection about 4 months prior. At that time, the patient was reported to have inappropriate urinary eliminations in the bed overnight and was scooting on the ground after being groomed. Physical examination revealed a two‐millimeter erosion on the right proximal labial fold, but was otherwise unremarkable. Urinalysis and sediment examination revealed an active sediment with 11‐20 white blood cells and 2‐3 red blood cells per HPF, 4‐10 struvite crystals per HPF, and >100 bacterial rods per HPF. The urine specific gravity was 1.028, urine pH was 8.5, and there was 1+ proteinuria on urine dipstick. *Escherichia coli* was cultured and was susceptible to multiple antibiotics. The patient was empirically prescribed Clavamox (amoxicillin and clavulanic acid) pending urine culture results, but the owners noted that the patient became pruritic and developed angioedema within 2 days of initiating the medication. Clavamox was discontinued due to suspected drug reaction, and marbofloxacin was prescribed pending the culture results. The cultured *E coli* was susceptible to both antibiotics. After completion of antibiotics, a repeat urine culture was negative for bacterial growth.

**Figure 1:**
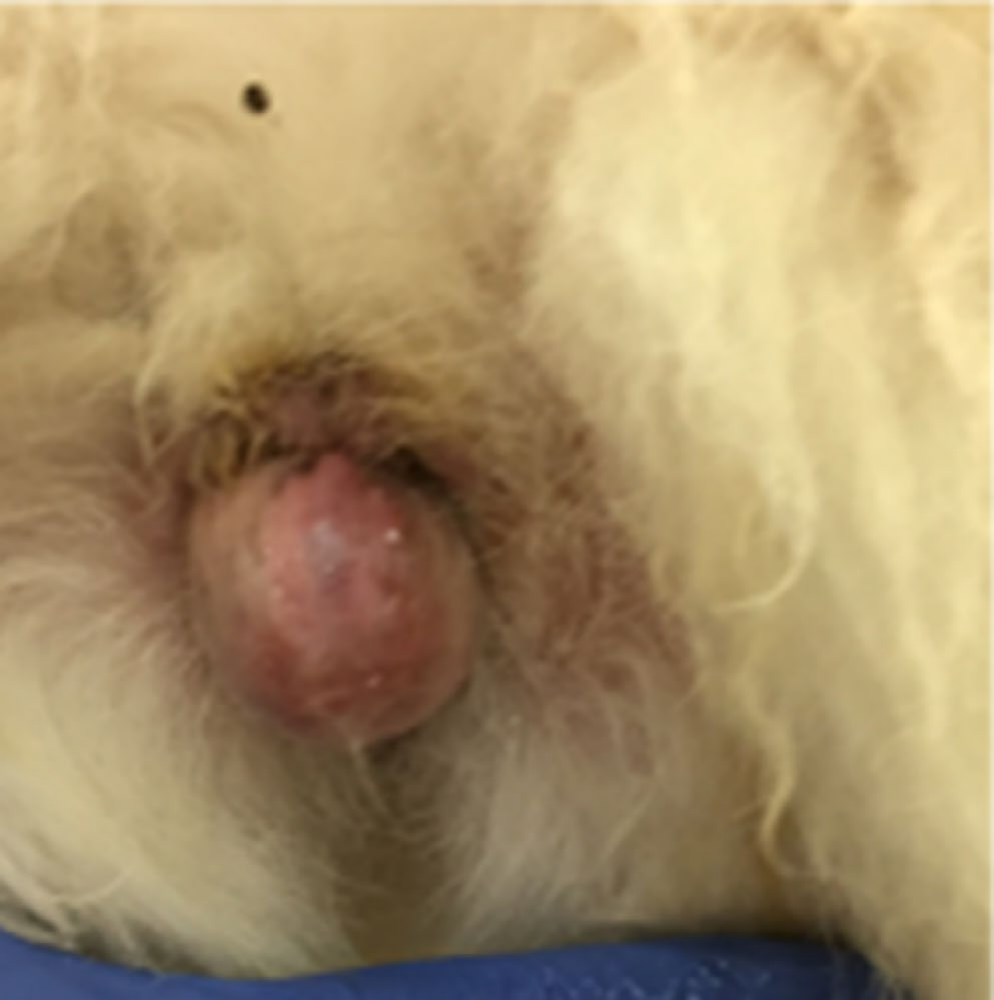
Image of an approximately 3.0 cm diameter, firm, hairless mass was protruding from the ventral commissure of the vulva

On physical examination at the authors' institution, an approximately 3.0 cm diameter, firm, hairless mass was noted protruding from the ventral commissure of the vulva (Figure 1). A fine needle aspirate of the mass was obtained; a clear, viscous fluid was visualized. The mass was noted to decrease in size immediately after aspiration, but partially refilled and remained soft in texture. Specific gravity of the aspirated fluid was 1.008, and cytologic findings were consistent with a cystic lesion with chronic lymphoplasmacytic inflammation, and no neoplastic cells or infectious agents were seen.

**Figure 2:**
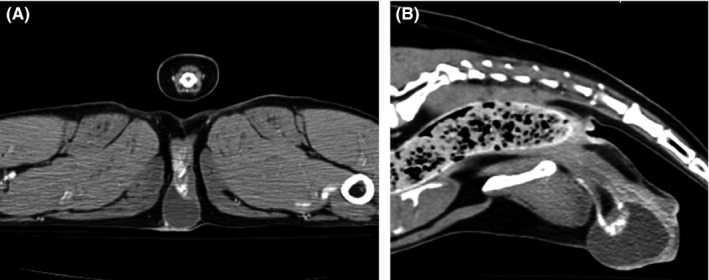
Transverse (A) and sagittal (B) postcontrast CT reconstructions. Arising from the ventral commissure of the vulva, in the region of the clitoris, there is a pedunculated thin‐walled fluid‐attenuating cystic mass. Dorsal to the mass, there is a nest of strongly contrast‐enhancing vessels that arise from clitoral and vulvar vasculature

Based on cytologic findings, a thoracoabdominal computed tomography (Computed tomography, Aquillion 64™; Toshiba) (CT) with iohexol (Ominpaque (iohexol injection); GE Healthcare AS) contrast was recommended to determine origin of the cyst and to aid in surgical planning. The CT was reviewed by a board‐certified veterinary radiologist and demonstrated a pedunculated, thin‐walled, fluid‐attenuating cystic mass, approximately 4.4 cm × 2.6 cm wide, which arose from the ventral commissure of the vulva in the region of the clitoral fossa and clitoris (Figure 2A). A nest of tortuous vasculature was present dorsal to the cystic component of the mass (Figure 2B,C). Additionally, there were several punctate mineral foci throughout the pulmonary parenchyma that were consistent with incidental heterotrophic bone, and the remainder of the CT scan was unremarkable with no evidence of lymphadenopathy or metastatic pulmonary disease.

**Figure 3:**
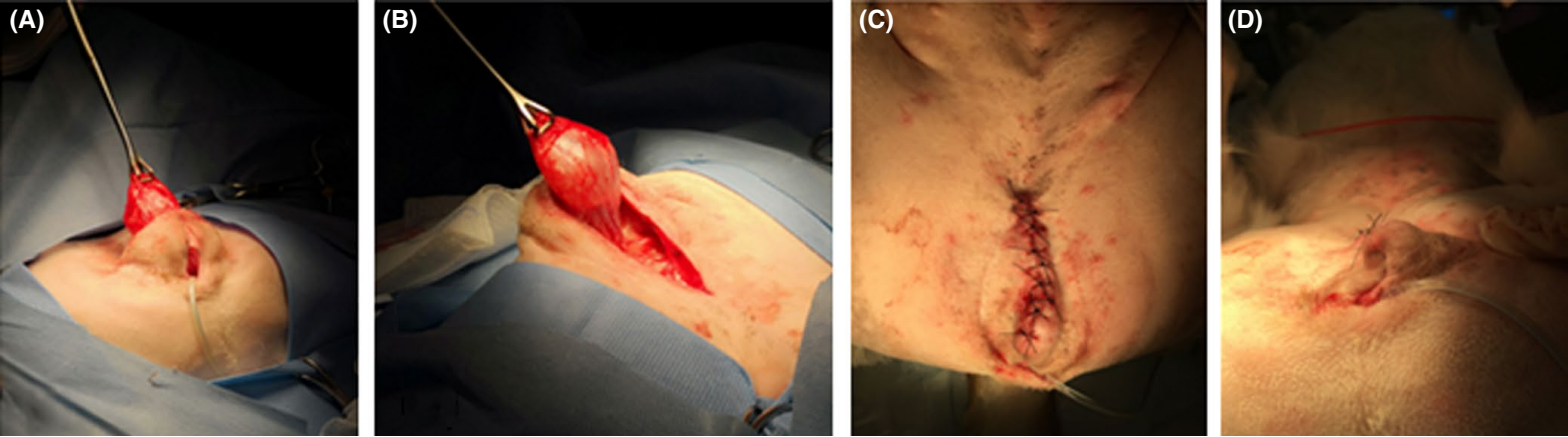
Imagines during surgery. View of clitoral mass and urethral catheter with patient in dorsal recumbency, draped for surgery (A). Clitoral mass exposed (B). Ventral view after surgical resection and reconstruction (C). Lateral view after surgical resection and reconstruction (D)

The patient was admitted to the hospital for surgical resection of the cystic structure. After premedication with dexmedetomidine (Dexdomitor; Zoetis, Orion Corporation) (5 mcg/kg IV) and hydromorphone (0.1 mg/kg IV), and induction with propofol (Propofol; Zoetis Inc) (6 mg/kg IV to effect), the patient was endotracheally intubated and general anesthesia was maintained with variable concentrations of isoflurane (Isoflurane; Patterson Veterinary) inhalant in 100% oxygen. Cefazolin (Cefazolin; Qilu Pharmaceutical Co., Ltd) (22 mg/kg IV every 90 minutes) was administered during the procedure. The patient was positioned in dorsal recumbency, with the pelvic limbs pulled forward. The perivulvar region was aseptically prepared, the patient was draped using aseptic technique, and an incision was made starting at the ventral commissure of the vulva and extended 8 cm cranially to expose the cystic mass. The cystic mass was found closely associated with the floor of the vagina in the region of the clitoris (Figure 3A). The mass was dissected using a combination of blunt and sharp dissection, and a combination of electrocautery (Valleylab force 1C electrosurgical generator; Valleylab) and 3‐0 Vicryl (Vicryl; Ethicon, Johnson Johnson Company) ligatures was used to achieve hemostasis (Figure 3B). An osclitoridis was identified, and a section of the ventral wall of the vulva, including the clitoris and osclitoridis, was removed along with the cystic mass. All excised tissues were submitted for histopathologic examination. The vaginal mucosa was apposed using 3‐0 Vicryl in a simple continuous pattern, the subcutaneous and subcuticular layers were apposed using 3‐0 Monocryl (Monocryl; Ethicon, Johnson Johnson Company) in a simple continuous pattern, and the skin was apposed using 3‐0 nylon (Nylon; Ethicon, Johnson Johnson Company) in a simple interrupted pattern (Figure 3C,D). The patient recovered uneventfully from general anesthesia. Postoperative treatments prescribed included a constant rate infusion of fentanyl (Fentanyl; Westward) (2 mcg/kg/h), cefazolin (22 mg/kg IV every 8 hours), and lactated ringer's solution (Lactated Ringer Solution; Braun Medical Inc) (60 mL/kg/d). Red‐tinged urine was noted several hours after surgery, but subsequently resolved without intervention. The patient was discharged the following day and was prescribed carprofen (Carprofen; Zoetis) (1.9 mg/kg by mouth every 12 hours for 14 days), codeine (Codeine; Westward) (1.2 mg/kg by mouth every 8 hours for 3 days), and cefpodoxime (Cefpodoxime; Sandoz) (7.7 mg/kg by mouth every 24 hours for 10 days).

**Figure 4:**
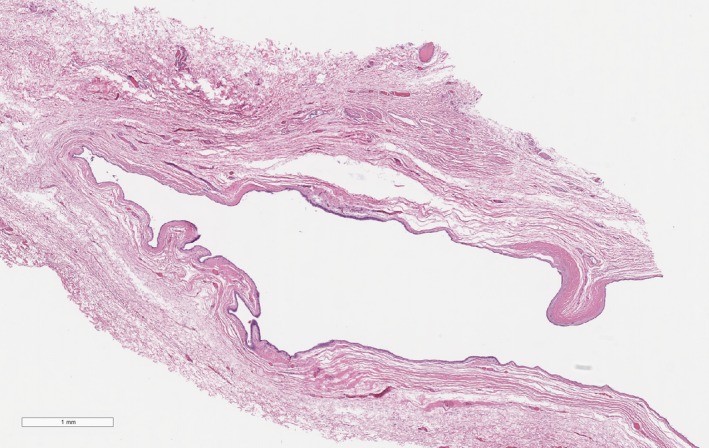
Histopathology of the excised clitoral cyst. Image credit: Antech Diagnostics and Barbara Powers, DVM, PhD, DACVP

**Figure 5:**
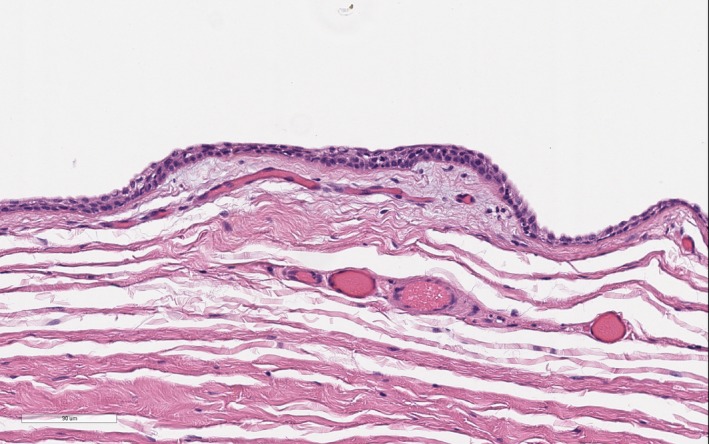
Histopathology of the excised clitoral cyst. Image credit: Antech Diagnostics and Barbara Powers, DVM, PhD, DACVP

The tissue submitted for histopathologic examination was standardly stained with hematoxylin and eosin and interpreted by a board‐certified pathologist. The microscopic diagnosis was an epithelial lined cyst with mild inflammation and no features of malignancy (Figure 4). The pathologist reported the tissue sections contained a large cystic structure devoid of contents. The wall was composed of cuboidal, to columnar, to pseudostratified columnar epithelial cells (Figure 5). Fibrosis was noted around the cyst wall, and within the fibrous tissue, there were areas of hypervascularity and a few scattered lymphocytes and plasma cells. The cyst was suspected to be completely excised based on the surrounding fibrous tissue.

Two days following discharge, the patient was re‐presented for evaluation of mild vaginal bleeding and discomfort. On presentation, the patient's vital parameters were within normal limits. Physical examination revealed the incision on the ventral aspect of the vulva was clean, dry, and intact, but that there was peri‐incisional bruising that extended to the caudoventral abdomen and inguinal region. A complete blood count showed a mild thrombocytopenia (126 k/μL; RI 148‐484 k/μL), but a manual blood smear showed an adequate platelet estimate; the rest of the results were within normal limits. The patient was admitted for overnight monitoring. Hydromorphone (Hydromorphone; Westward) (0.2 mg/kg IV once) was administered to facilitate vaginal examination. On vaginal examination, mild bleeding was noted. The perivulvar region was cleaned with sterile saline, and a generic tampon was inserted into the vaginal vestibule to achieve hemostasis. The following treatments were prescribed for overnight: cefpodoxime (7.6 mg/kg by mouth every 24 hours), codeine (1.7 mg/kg by mouth every 6 hours), trazodone (2.9 mg/kg by mouth every 8‐12 hours as needed for anxiety), application of a warm compress to the vulva for 5‐10 minutes every 6 hours, and change vaginal packing every 6 hours. The following morning, the patient was examined by the surgery service. Physical examination findings were similar to the previous evening. Ultrasound of the perivulvar region showed a fluid‐filled structure at the surgical site, which was suspected to either be a hematoma or a seroma. The surgeon recommended surgical exploration of the area for further evaluation. The patient was premedicated with dexmedetomidine (5 mcg/kg IV) and hydromorphone (0.1 mg/kg IV), was induced with propofol (3 mg/kg IV), and endotracheally intubated. General anesthesia was maintained with varying concentrations of isoflurane inhalant in 100% oxygen. The perivulvar region was aseptically prepared and draped, and a stab incision was made over the right cranial aspect of the previous incision. A large volume of liquid and clotted blood was evacuated, and the site was lavaged with sterile saline. A ¼ inch Penrose (Arglyepenrose tubing; Covidien) drain was placed into the hematoma and secured with single 3‐0 nylon suture. The patient recovered uneventfully from anesthesia and was discharged with instructions to continue the previously prescribed cefpodoxime, codeine, and carprofen as previously directed. Three days following discharge, the Penrose drain was removed, and the owners reported normal urinary function during a medical progress examination 3 months later.

## 3 DISCUSSION

The glans clitoridis is contained within the fossa clitoridis in the female dog.^1^ Differential diagnoses for anatomic abnormalities of the clitoris include an osclitoridis; clitoral, vaginal, vulvar, or urethral neoplasia; clitoral hypertrophy; pseudohermaphroditism; vaginal edema; vaginal prolapse; or a vaginal cyst.^7^, ^8^ Dogs with clitoral hypertrophy or an osclitoridis may be presented to a veterinarian for evaluation of a mass of tissue protruding from the vulva, excessive vulvar licking, or clinical signs associated with a urinary tract infection, such as pollakiuria, dysuria, stranguria, or hematuria.^2^, ^9^, ^10^ Not all dogs with clitoral abnormalities require treatment; however, when persistent clinical signs are noted, surgical treatment is generally most effective.^2^ Most commonly, either partial or complete clitorectomy is performed, which allows for removal of hypertrophied tissues and those clitoris, if present.^2^ In a case series evaluating 17 dogs diagnosed with an os clitoris, ten dogs underwent os clitorectomy.^2^ One dog was lost to follow‐up, but the other nine had resolution of associated clinical signs after healing.^2^ Similarly, neoplastic lesions, cysts, pseudohermaphroditism, and other clitoral or vaginal lesions may be managed surgically.^8^, ^9^, ^10^ Dogs have been surgically managed with osclitorectomy and resection of the hypertrophied clitoral tissue.^2^, ^9^ Dogs with female pseudohermaphroditism may present with dysuria, urinary incontinence, urine scald dermatitis, and recurrent urinary tract infections.^9^ Surgical intervention in these dogs can resolve the clinical signs. Clitoral carcinomas, though rare in dogs, may present with humoral hypercalcemia of malignancy and are associated with a poor prognosis.^8^ Dogs with vaginal cysts may present with dysuria, which may resolve with surgical resection.^11^

In humans, clitoral abnormalities are most frequently reported in association with female genital mutilation.^6^, ^12^, ^13^ Multiple types of cysts occurring in the clitoral region have been documented, including atheroma, dermoid cysts, dysontogenetic cysts, and epidermoid cysts.^6^ Although many cysts resolved spontaneously, surgical excision is sometimes pursued if the cyst is large or causing discomfort.^6^, ^12^ Patients are often asymptomatic when an abnormality is initially identified, but develop pain if there is associated inflammation or infection, may develop urinary incontinence or may have difficulty ambulating.^4^, ^14^ A variety of surgical techniques have been identified for excision.

Urinary incontinence is defined as involuntary urination during the storage phase of the urinary cycle.^15^ Clinically, this presents as intermittent or continuous dribbling of urine despite normal voiding.^15^, ^16^, ^17^ Differentials for urinary incontinence include urethral sphincter incompetence, anatomical abnormalities, inability of the bladder to dilate, spasms of the bladder, or damage to the nerves controlling micturition.^15^, ^16^, ^17^, ^18^, ^19^, ^20^, ^21^ In canines, urethral sphincter incompetence is the most common reason for urinary incontinence followed by ectopic ureter, detrusor instability, neurological, and uncommonly vaginal and rectal conditions.^15^, ^16^, ^17^, ^18^, ^19^, ^20^, ^21^ Urinary incontinence in humans has been associated with disorders of sexual development, primarily in those with ambiguous female external genitalia.^4^, ^14^ The exposed clitoris, when enlarged, may cause mechanical interference with the urethra, and contribute to urinary incontinence.^4^, ^14^ Similar mechanical interference has been described in cases of vaginal cysts.^22^, ^23^, ^24^ In one dog that presented with an osclitoridis, there may have been derangements of normal urethral anatomy, including the urethra coursing through the hypertrophied clitoris.^23^ A definitive mechanism for this patient's urinary incontinence was not identified, but clinical signs resolved with removal of the mass.^23^

In conclusion, patients presenting for lower urinary tract symptoms, such as excessive vulvar licking, urinary incontinence, or recurrent urinary tract infections, should have a thorough external genital examination. If a clitoral cyst is suspected, surgical excision may be curative.

## CONFLICT OF INTEREST

None declared.

## AUTHOR CONTRIBUTIONS

CF: primary manuscript author. JO: heavily involved with manuscript editing. MG: manuscript editor.
